# Invasive Pneumococcal Diseases Before and After the COVID-19 Pandemic in Italy (2018–2023)

**DOI:** 10.3390/microorganisms13122734

**Published:** 2025-11-30

**Authors:** Romina Camilli, Sara Giancristofaro, Stefano Boros, Benedetta Bellini, Fabio D’Ambrosio, Roberta Urciuoli, Maria Del Grosso, Annalisa Pantosti, Anna Teresa Palamara, Fortunato D’Ancona

**Affiliations:** Department of Infectious Diseases, Istituto Superiore di Sanità, 00161 Rome, Italy

**Keywords:** *Streptococcus pneumoniae*, vaccines, pneumococcal serotypes, invasive pneumococcal diseases, COVID-19, Italy

## Abstract

This study assessed the epidemiological and microbiological invasive pneumococcal disease (IPD) changes that occurred before and after the emergence of COVID-19 in Italy. All IPD cases reported through the nationwide surveillance system during 2018–2023 were included. IPD incidence and serotype distributions were analyzed by age group. IPD incidence in 2020–2021 declined in all age groups compared with 2018–2019, especially in children less than 2 years of age and elderly people aged > 64 years. A resurgence of IPD cases was observed from late 2022 onwards, with values in children exceeding those seen before the pandemic. The post COVID-19 increase in children was mainly driven by some PCV13 serotypes, such as 3, 19A, and 19F, but also non-vaccine serotypes, including 10A, 8, and 24F, while in the elderly population, a predominance of serotypes 3 and 8 was observed. In conclusion, a steep drop in IPD incidence was observed during the peak of the COVID-19 pandemic, followed by a subsequent upsurge of cases, especially in children. Continuous national surveillance is necessary to monitor the dynamics and evolution of IPD and the impact of new higher-valency vaccines in Italy over the next few years.

## 1. Introduction

Invasive pneumococcal disease (IPD) is associated with high morbidity and mortality rates worldwide, especially in the two high-risk age groups: children under 5 years of age and the elderly. The introduction of pneumococcal conjugate vaccines (PCVs) profoundly changed the epidemiology of IPD in countries where these vaccines were used, leading to a reduction in PCV-serotype IPD incidence not only in vaccinated children but, due to herd protection, also in older unvaccinated individuals [1]. However, the success of PCVs was impaired by a progressive increase in IPD incidence caused by non-vaccine serotypes (NVS) [2,3].

In Italy, the 7-valent PCV (PCV7, including serotypes 4, 6B, 9V, 14, 18C, 19F, 23F) was replaced by the 13-valent PCV (PCV13, including additional serotypes 1, 3, 5, 6A, 7F, and 19A) in 2010. Universal pediatric vaccination was recommended at national level in the 2012–2014 National Immunization Plan, which offered PCV vaccination to all newborns according to the 2 + 1 doses at 3, 5 and 11 months of age, and to children less than 5 years of age who had missed the doses during their first year of life. Subsequently, in the 2017–2019 National Immunization Plan, pneumococcal vaccination was also recommended for individuals aged over 64 years and for all individuals at increased risk of invasive pneumococcal disease, following the sequential administration of PCV13 and of the 23-valent polysaccharide vaccine PPSV23 one year apart [4]. Over the years, the national PCV coverage, assessed at 24 months for the completed cycle, progressively increased, reaching 91.73% in 2022 (2020 birth cohort) [5].

As in other countries, the introduction of PCVs led to significant changes in IPD in Italy. A study from our group on IPD in seven Italian regions during 2008–2014 reported a 56% decline in overall IPD incidence in children less than 5 years of age, mainly attributable to the decrease in cases in the first year of life [6]. A similar decline in IPD incidence (61%) was observed in the same age group in another study focused on Northern Italy during the same period [7].

In 2022, new higher-valence glycoconjugate vaccines were authorized—a 15-valent PCV (PCV15, including additional serotypes 22F and 33F) and a 20-valent PCV (PCV20, including additional serotypes 8, 10A, 11A, 12F, and 15B), which are currently available nationwide for immunization of children and adults. More recently, a 21-valent PCV (PCV21) with a different serotype selection was authorized for vaccination in adults.

To cope with the COVID-19 pandemic in 2020, several non-pharmacological interventions (NPIs) such as social distancing, face masks, and lockdowns were implemented in the Italian population. These measures not only reduced the transmission of SARS-CoV-2, but also that of other respiratory infections, including those caused by pneumococcus [8]. The decline in IPD cases during this time was likely also affected by organizational factors such as reduced laboratory testing capacity and limited capacity for surveillance and case reporting [9]. In addition, some studies have investigated the role of respiratory viruses in the etiology of IPD during and after the COVID-19 pandemic, highlighting a causal link between viral infection and the onset of IPD [10,11].

In this study, we present an overview of epidemiological and microbiological changes in IPD incidence and circulating serotypes over a 6-year period that includes the emergence of COVID-19 in Italy.

## 2. Materials and Methods

### 2.1. Study Design

In Italy, a surveillance system for IPD, coordinated by the National Institute of Health (Istituto Superiore di Sanità, ISS), has been in place since 2007. The surveillance system collects data on IPD cases that have been laboratory-confirmed by culture and/or molecular methods, i.e., detection of pneumococcal DNA in cerebrospinal fluid, blood, or other normally sterile sites. Reported data included demographic information, clinical presentation, and laboratory results. Clinical presentations included meningitis (with or without associated sepsis/bacteremia), sepsis/bacteremia, pneumonia, and other manifestations, such as endocarditis, arthritis, or empyema, with diagnoses confirmed in blood or other sterile sites (i.e., endocardial fluid, synovial fluid, pleural fluid).

All IPD cases reported in the surveillance system during 2018–2023 were included in this observational retrospective study.

### 2.2. Bacterial Strains and Serotyping

*S. pneumoniae* strains were cultured on 5% Columbia sheep blood agar plates and incubated overnight at 37 °C in 5% CO_2_-enriched air. Serotyping of pneumococcal strains was performed either at the regional reference laboratories or at the national reference laboratory (NRL) at ISS by Latex agglutination, Quellung reaction using antisera provided by SSI Diagnostica (HillerØd, Denmark) or by molecular methods [12,13,14].

### 2.3. Statistical Analysis

IPD annual incidence was calculated using the number of cases reported to the surveillance system during 2018–2023 and the estimated population for each age group on 1st January of each year, according to the National Institute for Statistics, http://demo.istat.it (accessed on 16 October 2024). Differences in incidence were calculated using Poisson regression models as (1 − IRR) × 100, where IRR is the incidence rate ratio, adjusted for regional PCV coverage. A multivariable logistic regression analysis was performed to assess randomness for missing serotyping data. Statistical analyses were performed using R software (version 4.5.0).

## 3. Results

### 3.1. IPD Incidence

Between 2018 and 2023, a total of 7072 IPD cases were reported in the IPD surveillance system, including 311 cases (4.4%) from children under 5 years and 4187 cases (59.2%) from adults aged > 64 years. Among all IPD cases, 5399 (76.3%) were diagnosed using culture methods, 839 (11.86%) with molecular techniques, and 834 (11.8%) using a combination of both approaches.

During the full COVID-19 pandemic years of 2020–2021, an overall sharp decline in IPD incidence was observed for all age groups compared to the previous two years, with incidence rate ratios (IRR) of 0.78 (95% CI; 0.42–1.44) in children under 2 years, 0.44 (95% CI; 0.18–1.03) in children 2–4 years old, 0.33 (95% CI; 0.29–0.36) in adults 18–64 years old and 0.3 (95% CI: 0.28–0.33) in adults aged >64 years (Table 1).

**Figure 1:**
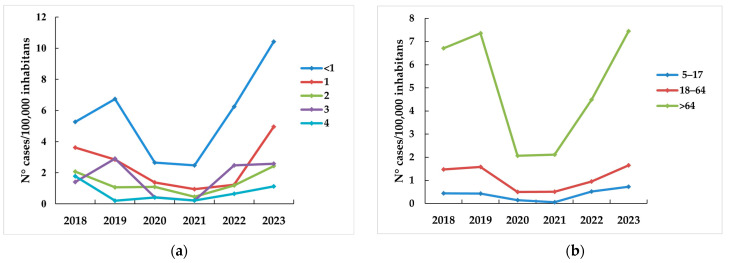
IPD incidence (N° cases/100,000 inhabitants) by age group during 2018–2023 in Italy. (**a**) IPD incidence in children <1, 1, 2, 3, and 4 years old. (**b**) IPD incidence by age group (5–17, 18–64, and > 64 years old).

**Figure 2:**
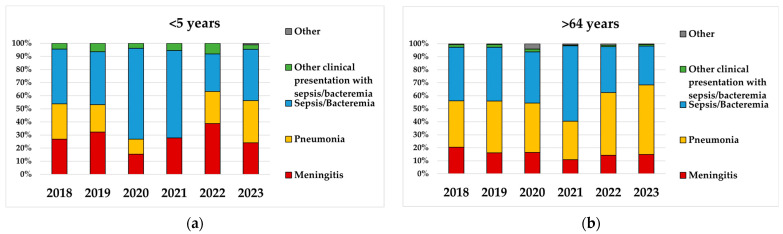
Clinical presentations by age group during 2018–2023 in Italy. (**a**) Children aged <5 years. (**b**) Adults aged >64 years.

Over the next two years, however, a resurgence in IPD cases was observed, reaching or exceeding the pre-pandemic levels in 2023 (Table 1, Figure 1). Notably, IPD incidence in children less than 1 year old reached 10.4 cases/100,000 inhabitants in 2023, much higher than in 2019 (6.7 cases/100,000 inhabitants), while the IPD incidence in children aged 1 year almost doubled (2.9 cases vs. 5 cases/100,000 inhabitants) (Figure 1). For adults aged >64 years, IPD incidence decreased in 2020–2021 to about 2 cases/100,000 inhabitants and then increased again to pre-pandemic levels in 2023 (7.5 cases /100,000 inhabitants) (Figure 1). Interestingly, in 2023, unlike previous years when sepsis was more commonly reported, bacteremic pneumonia became the most common clinical presentation in both children and adults (Figure 2).

**Figure 3:**
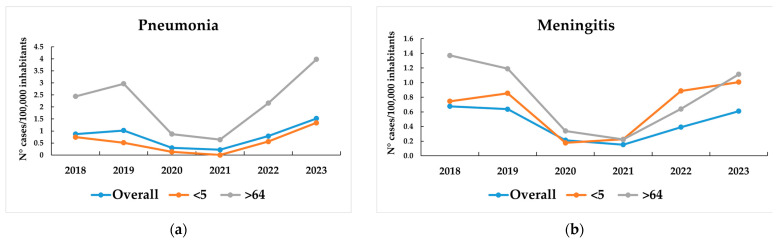
Incidence of pneumonia and meningitis cases (N° cases/100,000 inhabitants) by age group during 2018–2023 in Italy. (**a**) Incidence of pneumonia cases in children aged <5 years, adults aged >64 years, and the overall population. (**b**) Incidence of meningitis cases in children aged <5 years, adults aged >64 years, and the overall population.

In children < 5 years, the incidence of bacteremic pneumonia rose from 0.75 cases/100,000 inhabitants in 2018 to 1.3 in 2023 (18 cases and 28 cases, respectively), while in adults aged > 64 years, it increased from 2.4 cases/100,000 inhabitants in 2018 to 4 in 2023 (331 cases and 564 cases, respectively) (Figure 3). Regarding meningitis, incidence declined in 2020–2021 but subsequently increased across all age groups; in children, incidence exceeded pre-pandemic levels, reaching 1 case/100,000 inhabitants in 2023 (Figure 3).

### 3.2. Serotype Distribution in Children <5 Years and Adults Aged >64 Years

**Figure 4:**
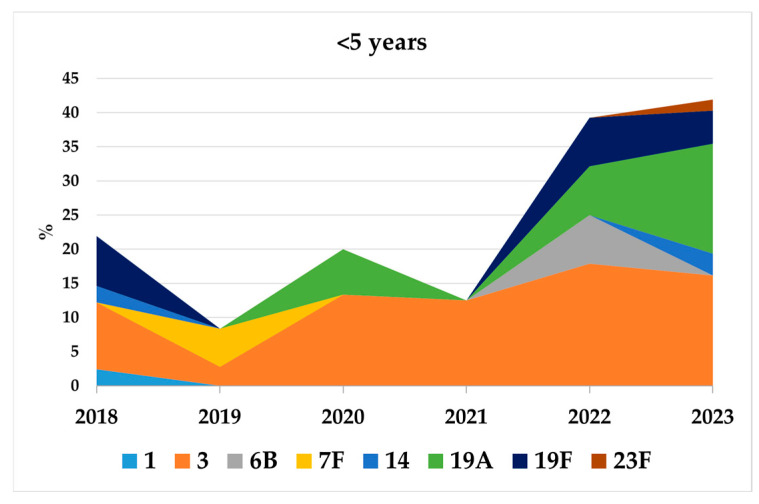
Proportion in percentage of individual PCV13 serotypes (serotypes 1, 3, 6B, 7F, 14, 19A, 19F, and 23F) recovered in IPD cases in children < 5 years old during 2018–2023.

Serotyping was available for 4430 out of 7072 cases (62.64%) reported in the surveillance system during 2018–2023. A multivariable logistic regression analysis showed that completeness was slightly lower during the pandemic and in southern regions (Table S1). After the COVID-19 pandemic, an increase in the incidence of IPD due to PCV13 serotypes was observed in 2022–2023 in all age groups, particularly in children under 2 years of age (IRR: 2.48; 95% CI: 0.92–6.70) and 2–4 years (IRR: 3.37; 95% CI: 1.06–10.70) (Table 1). Among children <5 years, the proportion of PCV13-serotype IPD, which had declined in 2018–2019, doubled from 21.4% in 2018 to 41.9% in 2023. The increase in 2023 was mainly driven by serotypes 3 and 19A (16.1% each) and serotypes 19F (4.8%) and 14 (3.2%) (Figure 4).

**Figure 5:**
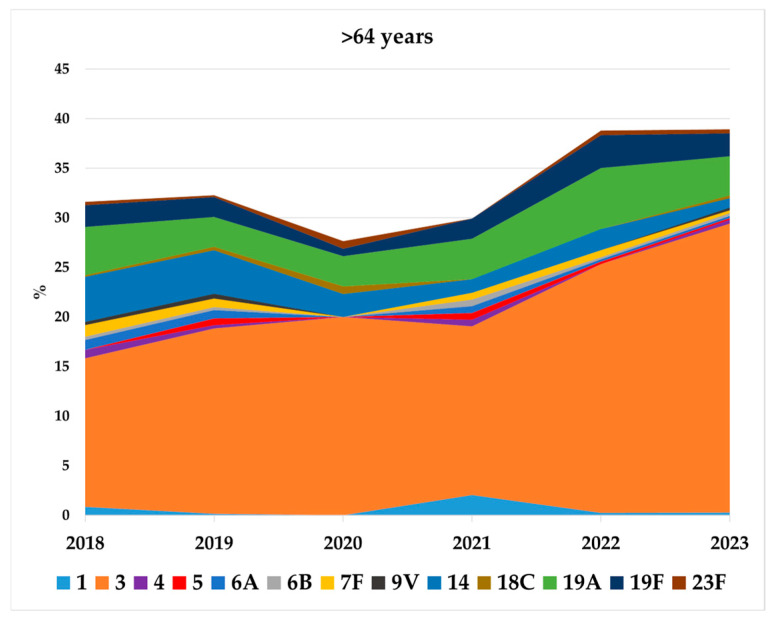
Proportion in percentage of individual PCV13 serotypes (serotypes 1, 3, 4, 5, 6A, 6B, 7F, 9V, 14, 18C, 19A, 19F, and 23F) recovered in IPD cases in adults >64 years old during 2018–2023.

In adults >64 years old, PCV13 serotypes increased during 2022–2023 but to a lesser extent (IRR: 1.21; 95% CI: 1.1–1.33), rising from 32% in 2018 to 39% in 2023. No increase was observed during the same period for PPV23 serotypes (IRR: 0.72; 95% CI: 0.66–0.79) or additional PPV23 serotypes (IRR: 0.66; 95% CI: 0.59–0.73) (Table 1). In this age group, serotype 3 remained the most common, accounting for 29.2% of overall IPD cases in 2023, followed by serotypes 19A, 19F, and 14, which together represented 7.2% of cases (Figure 5).

**Figure 6:**
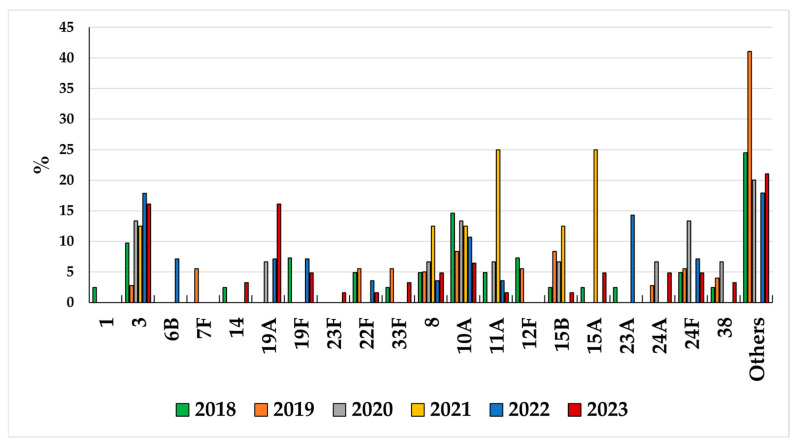
Serotype distribution in children < 5 years old during 2018–2023.

Concurrently, in the post-COVID-19 years, IPD cases caused by non-PCV13 serotypes increased in children < 2 years (IRR: 1.35; 95% CI: 0.85–2.13) (Table 1). Overall, in children < 5 years of age, the most common non-PCV13 serotypes in the post-pandemic period were, in decreasing order, serotypes 10A, 8, 24F, 15A, 38, 11A, and 15B (Figure 6).

**Figure 7:**
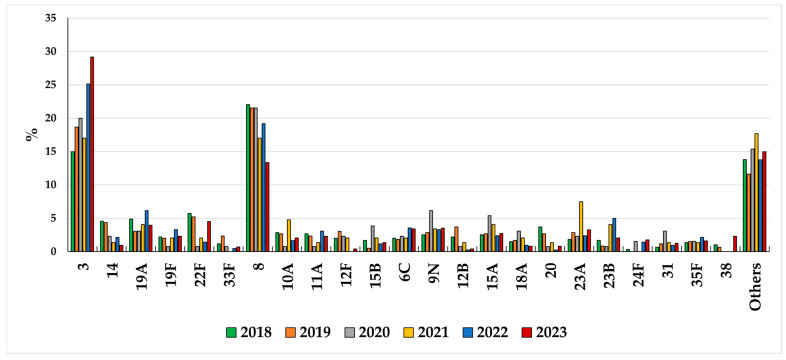
Serotype distribution in adults > 64 years old during 2018–2023.

In adults aged > 64 years, serotype 8 was the most common among non-PCV13 serotypes from 2018 to 2020 and the second most common in 2021–2023, accounting for 13.4% of overall IPD in 2023 (Figure 7).

Several other non-PCV13 serotypes were distributed in this age group, including 22F, 6C, 9N, 23A, 15A, 38, 11A, 10A, 23B, 24F, 35F, and 15B, although their frequencies varied from year to year (Figure 7). Indeed, serotypes 3 and 8 together were the predominant cause of IPD in individuals aged > 64 years throughout the entire period, rising from 37% in 2018 to 43% in 2023.

Between 2018 and 2023, nine serotypes were responsible for the majority (≥60% cases) of bacteremic pneumonia cases in adults aged > 64 years, listed in decreasing order serotypes 3, 8, 19A, 22F, 9N, 6C, 23A, 23B, and 19F, with the first two accounting for 48% of total cases in 2023 (Figure S1). Among cases of bacteremic pneumonia in children, serotype 3 was the most frequently detected, accounting for 26% of cases in 2023 (Figure S2). Regarding serotype distribution in meningitis cases, serotypes 10A, 19F, 19A, and 23B were responsible for 43% of overall cases in children in 2022–2023, while in adults aged >64 years, the most prevalent serotypes during the same period were, in decreasing order, 3 (16%), 8 (12%), 6C (9%), 19F (7%), 10A and 23A (6% each) (Figures S3 and S4).

### 3.3. Serotype Distribution by New Conjugate Vaccines in Children <5 Years and Adults Aged >64 Years

**Figure 8:**
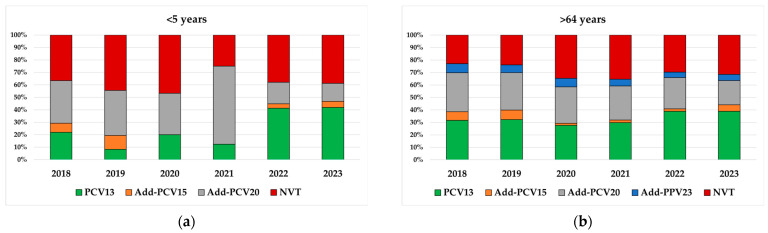
Serotype distribution according to PCV13 serotypes (1, 3, 4, 5, 6A, 6B, 7F, 9V, 14, 18C, 19A, 19F, 23F), PCV15 additional serotypes (22F and 33F), PCV20 additional serotypes (8, 10A, 11A, 12F, and 15B), PPV23 additional serotypes (2, 9N, 17F, and 20 minus 6A) and non-vaccine type (NVT), 2018–2023. (**a**) Serotype distribution in children <5 years. (**b**) Serotype distribution in adults >64 years.

The proportion of IPD cases potentially covered by new conjugate vaccines, such as the recently licensed PCV15 and PCV20, or the upcoming PCV21 and PCV24, was evaluated in both children < 5 years old and adults > 64 years old. The frequency of PCV15 serotypes reached 47% in children and 44% in adults in 2023, with about 5% more serotypes covered in both age groups than with PCV13 in the last year (Figure 8). PCV20-serotype IPD represented around 61% of cases in children, 15% more than PCV15 in 2023, with no major temporal changes over the study period (Figure 8). Similarly, among adults aged >64 years, PCV20 would have covered 64% of cases in 2023, 20% more than PCV15 (Figure 8). The potential coverage increased further with PCV21 and PCV24, reaching 71% and 66% of cases in children and 75% and 69% in adults, respectively, in 2023 (Figure S5).

## 4. Discussion

This study examined IPD trends in the pre- and post-COVID-19 pandemic eras. In line with other countries worldwide [15], a prompt decline in IPD was also documented in Italy during the peak of the COVID-19 pandemic in 2020–2021, with an overall 67% reduction and a substantial decline in children under 2 years of age and adults aged >64 years. The implementation of measures such as NPIs to counter SARS-CoV-2 transmission has indirectly led to a decrease in other respiratory infections, either viral or bacterial, including pneumococcal infections. Recent studies suggest that the concurrent decline in viral respiratory infections, such as those caused by influenza or respiratory syncytial virus (RSV), was an important factor in the reduction in pneumococcal infections, rather than decreased carriage [10,16]. Over the last four months of 2022, a gradual increase in IPD incidence was observed, likely due to the relaxation of the containment measures and the restoration of the ecological dynamics of the commensal and pathogenic flora. Notably, in the last year under study, the incidence in children under 1 year reached a level never recorded in pre-pandemic years, while in adults aged >64 years it returned to pre-pandemic levels. The resurgence of invasive bacterial infections with respiratory transmission, such as IPD, after the COVID-19 pandemic has been documented worldwide, albeit with varying timing and magnitude [17,18]. Some authors have proposed that the rebound in IPD, particularly in children, may partly result from an “immune debt” incurred by the population due to reduced pathogen exposure during pandemic restrictions, thereby increasing disease susceptibility in high-risk groups, such as newborns [19,20,21]. In addition, the resurgence of viral infections, such as RSV, could have contributed to the increase in IPD [11,22,23,24]. Notably, our dataset showed an increase in bacteremic pneumonia cases in both children and adults in 2023. The extent to which this reflects a true rise in incidence, instead of biases such as improved post-COVID diagnostic capacity, remains to be determined. However, a recent study by Cocchio et al., investigating the pneumonia-related hospitalizations among the elderly in an Italian region, showed a significant positive trend for pneumococcal pneumonia in 2023 [25]. Moreover, since the COVID-19 pandemic, other studies have reported a substantial increase in pneumonia cases, both bacterial and viral, invasive and non-invasive [26,27]. When analyzing the distribution of serotypes, an overall increase in PCV13 serotypes was observed alongside the rise in cases during 2022–2023, particularly among children under 5 years of age and, to a lesser extent, among adults aged >64 years. In children, this increase was mainly driven by serotypes 3 and 19A, followed by serotypes 19F and 14, while serotype 3 was more common in adults aged >64 years. Interestingly, although more than a decade has passed since the introduction of PCV13 for childhood immunization, an increase in the proportion of PCV13 serotypes among European countries during 2018–2022 was described in the most recent ECDC annual epidemiological report on IPD, confirming the predominance of serotypes 3 and 19A in children and of serotype 3 in adults [28]. A post-pandemic increase in PCV13-type IPD, mainly due to serotype 3, but also serotypes 19F, 19A, and 4, was also recently described in England [29]. During the pandemic, healthcare services, including those responsible for vaccinations, were overwhelmed, which may have affected the completion of and adherence to the routine PCV vaccination schedule. However, this issue cannot be generalized, and some countries reported a decline in vaccination coverage [30,31] whereas others even observed an increase [32]. In Italy, during 2020–2022, there was only a slight decrease in national PCV coverage (assessed at 24 months) compared to 2019 (92%), with the lowest value recorded in 2020 (90.58%). Still, it increased again in the following two years (91.25% and 91.73%, respectively) [5].

The low effectiveness of PCV13 against serotype 3 is well documented worldwide, mainly due to the unique structure of the capsular polysaccharide. Type 3-polysaccharide, due to its lack of covalent binding to the bacterial surface, is released in substantial amounts during an infection. This release shields the bacterial surface from the effects of vaccine-induced antibodies, ensuring bacterial survival. In addition, several studies have shown that PCV13 provided short-lived protection against type 3 polysaccharide [33,34] and insufficient herd immunity [35,36]. Notably, serotype 3 was the primary cause of bacteremic pneumonia in children and adults in our case series. This is consistent with other studies investigating the role of serotype 3 in complicated pneumonia or adverse cardiac events [27,37]. A recent paper from Lodi et al. [13], focusing on serotype 3 IPD in an Italian region, showed a correlation between serotype 3 and bacteremic pneumonia or otomastoiditis, but not with sepsis or meningitis, supporting the hypothesis that PCV13 could prevent only the more disseminated forms of IPD [38,39,40]. Interestingly, the authors showed a strong temporal association between serotype 3 IPD and peaks of influenza virus, but not with peaks of RSV, especially post-COVID-19 pandemic. Serotype 19A is another PCV13 serotype on the rise in our country, as well as in other European countries, following the pandemic. Although this serotype showed a downward trend in children after PCV13 introduction in many countries, including Italy [3,6,7], it is one among the vaccine’s serotypes that persisted and is most frequently associated with vaccine failure/breakthrough cases, likely due to waning immunity at 12 months of age [41,42]. In Australia, three years after PCV13 introduction, serogroup 19 remained an important cause of pediatric IPD. It was hypothesized that reduced vaccine efficacy may result from heterogeneity in the capsular polysaccharide locus of serogroup 19, suggesting that immune responses to 19F and 19A strains used in PCVs may not protect against all serogroup 19 strains [43]. More recently, a study from Ireland examined the persistence of serotype 19A and highlighted that vaccine failures were associated with the expansion of a sub-clade characterized by mutations in *galE*, a gene involved in capsule production [44].

Considering non-PCV13 serotypes, in children aged less than 5 years, serotypes 10A, 8, and 24F were among the most prevalent during the entire period under study. Serotypes 10A and 24F are typically associated with diseases in the pediatric population, and in our dataset, serotype 10A is among the major causes for meningitis cases in recent years. A recent study from Argentina on meningitis trends over ten years post PCV implementation described an increase in the proportions of serotype 10A and serogroup 24, along with serotypes 15B/C in children [45]. Serotype 24F has a high invasive potential and is currently reported to be one of the most prevalent serotypes in children, not only in European countries [28,46,47,48] but also in other continents [49]. In recent years, as in other European countries, serotype 8 has become the most prevalent non-PCV13 serotype in Italy, especially in the elderly, but also in children. Serotype 8, like serotypes 3 and 37, is characterized by a highly mucoid-type capsule, suggesting greater virulence capacity. Indeed, a recent study by Perez-Garcia et al. [50] demonstrated that the increase in serotype 8 is associated with lineages and mutations in the capsular operon that have different potential to cause IPD. The uneven replacement of serotype 8 in certain countries post PCV13 introduction could be attributed to the prevalence of lineages with lower disease potential.

As of 2022, a new series of higher-valency PCVs (PCV15, PCV20 and more recently PCV21) have been licensed and introduced for the immunization of children and adults, while other novel PCVs with a broader spectrum, such as PCV24, are in various developing stages. These vaccines expand the number of covered serotypes and are expected to cope with the replacement of some non-PCV13 serotypes. However, it will be interesting to assess PCV15 efficacy against serotype 3, given the higher anti-serotype 3 titers reported in pre-licensure studies compared to PCV13, which could result in more durable protection [51,52]. Based on our serotype prevalence, PCV15 could have covered an estimated 5% of additional cases, both in children and adults, with respect to PCV13 in 2023. Regarding PCV20, the percentage of covered cases reached more than 60% in both children and adults in 2023, while for PCV21, recommended only for adults, the percentage increased to 75%. These data establish an essential national baseline for monitoring and assessing the future impact of these higher-valency vaccines in Italy.

This study has several potential limitations to consider. Our surveillance system collects IPD cases at the national level. Still, since it is not mandatory and regional differences in case reporting were observed, some underestimation of cases may have affected our analysis. Another limitation is the lack of studies examining potential changes over time of testing capacity, clinical practices such as increased use of molecular diagnostics and altered thresholds for blood culturing, or heightened awareness of respiratory pathogens that may have contributed to an enhanced detection of IPD and bacteremic pneumonia cases after the pandemic. Additionally, serotyping data were available for about 64% of cases, with a slightly higher proportion of missing data during the pandemic and in southern regions, suggesting variations in regional surveillance adherence that may have influenced the observed serotype distribution. However, despite these potential limitations, our results align with several other European countries regarding IPD incidence and serotype trends.

## 5. Conclusions

In conclusion, this observational study documented a rapid decline in IPD incidence across all age groups nationwide during the pandemic, likely due to the implementation of NPIs that reduced the circulation of pathogenic bacteria and viruses. With the easing of NPIs, increases in respiratory infectious diseases were observed, including IPD, which returned to pre-pandemic levels or higher. Notably, after the pandemic, a rise in some PCV13 serotypes was recorded. This increase involved not only serotype 3, for which PCV13 was of limited efficacy, but also other serotypes, such as 19A and 19F, which had previously declined. At the same time, several other non-PCV13 serotypes increased, some of which are included in the new PCVs. These findings underline the importance of enhanced, continuous national surveillance to verify the persistence of specific serotypes, monitor serotype replacement, and assess the impact of new vaccines on the burden of IPD in Italy.

## Figures and Tables

**Table 1:** Number of cases and incidence of IPD (N° cases/100,000 inhabitants) by age group in 2018–2019, 2020–2021, and 2022–2023. Incidence rate ratios (IRR), adjusted for regional PCV coverage for age groups <2 years old and 2–4 years old, were estimated from Poisson regression models comparing the different time periods according to age group and vaccine serotypes PCV13 (1, 3, 4, 5, 6A, 6B, 7F, 9V, 14, 18C, 19A, 19F, 23F), PPV23 (1, 2, 3, 4, 5, 6B, 7F, 8, 9N, 9V, 10A, 11A, 12F, 14, 15B, 17F, 18C, 19A, 19F, 20, 22F, 23F, 33F), additional PPV23 (2, 8, 9N, 10A, 11A, 12F, 15B, 17F, 20, 22F, 33F minus 6A) and non-vaccine type (NVT).

	2018–2019		2020–2021		2022–2023				
	Cases (N°)	Incidence /100,000	Cases (N°)	Incidence /100,000	Cases (N°)	Incidence /100,000	IRR 2020–2021 vs. 2018–2019 (95% CI)	IRR 2022–2023 vs. 2018–2019 (95% CI)	IRR 2022–2023 vs. 2020–2021 (95% CI)
**<2 years old**	83	4.6	31	1.9	91	5.7	0.78 (0.42–1.44) *	1.40 (0.93–2.11) *	1.79 (1.03–3.13) *
PCV13	6		2		18		1.72 (0.34–8.67) *	2.48 (0.92–6.70) *	1.44 (0.33–6.26) *
Non-PCV13	43		14		44		0.59 (0.30–1.15) *	1.35 (0.85–2.13) *	2.29 (1.24–4.21) *
**2–4 years old**	46	1.6	13	0.46	45	1.7	0.44 (0.18–1.03) *	0.97 (0.56–1.68) *	2.22 (1.04–4.71) *
PCV13	6		2		20		0.68 (0.09–4.85) *	3.37 (1.06–10.70) *	4.98 (1.09–22.77) *
Non-PCV13	26		7		15		0.47 (0.19–1.20) *	0.56 (0.29–1.12) *	1.19 (0.48–2.97) *
**18–64 years old**	1.116	1.5	365	0.50	928	1.3	0.33 (0.29–0.36)	0.83 (0.78–0.89)	2.54 (2.38–2.71)
PCV13	186		59		245		0.32 (0.24–0.41)	1.32 (1.16–1.49)	4.15 (3.65–4.71)
PPV23	477		198		316		0.42 (0.36–0.48)	0.66 (0.59–0.74)	1.6 (1.42–1.78)
Add-PPV23	282		71		235		0.25 (0.20–0.32)	0.83 (0.73–0.95)	3.31 (2.9–3.76)
NVT	171		37		132		0.22 (0.15–0.3)	0.77 (0.65–0.92)	3.57 (2.98–4.23)
**>64 years old**	1.918	7.0	581	2.1	1.687	6.0	0.30 (0.28–0.33)	0.88 (0.84–0.92)	2.9 (2.77–3.05)
PCV13	369		79		447		0.21 (0.17–0.27)	1.21 (1.1–1.33)	5.66 (5.15–6.21)
PPV23	741		305		535		0.41 (0.37–0.46)	0.72 (0.66–0.79)	1.75 (1.61–1.91)
Add-PPV23	530		100		349		0.19 (0.15–0.23)	0.66 (0.59–0.73)	3.49 (3.13–3.88)
NVT	278		97		356		0.35 (0.28–0.43)	1.28 (1.15–1.42)	3.67 (3.3–4.07)
**TOTAL**	3.226	3.1	1.005	0.96	2.839	2.7	0.31 (0.29–0.33)	0.88 (0.85–0.91)	2.82 (2.72–2.93)
PCV13	591		144		756		0.24 (0.21–0.29)	1.28 (1.19–1.37)	5.25 (4.88–5.64)
Non-PCV13	1396		337		1208		0.24 (0.22–0.27)	0.87 (0.82–0.92)	3.58 (3.39–3.79)

* Adjusted for regional PCV coverage.

## Data Availability

The original contributions presented in this study are included in the article. Further inquiries can be directed to the corresponding author.

## Supplementary Materials

The following supporting information can be downloaded at: https://www.mdpi.com/article/10.3390/microorganisms13122734/s1, Table S1: Multivariable logistic regression assessing factors associated with missing serotype data among invasive pneumococcal disease (IPD) cases, Italy, 2018–2023; Figure S1: Serotype distribution in pneumococcal pneumonia in adults > 64 years of age, 2018–2023; Figure S2: Serotype distribution in pneumococcal pneumonia in children <5 years of age in 2023; Figure S3: Serotype distribution in pneumococcal meningitis in children <5 years of age, 2022–2023; Figure S4: Serotype distribution in pneumococcal meningitis in adults >64 years of age, 2022–2023; Figure S5: Serotype distribution by PCV21 and PCV24 in IPD in children <5 years and adults >64 years old in 2023.
