# Supplementary material for: Invasive Pneumococcal Diseases Before and After the COVID-19 Pandemic in Italy (2018–2023)
Source: Microorganisms. 2025 Nov 30;13(12):2734. doi: 10.3390/microorganisms13122734 (PMC12735837; doi:10.3390/microorganisms13122734)
Supplement: Supplementary file 1 [file microorganisms-13-02734-s001.zip › Supplementary_Materials_ Table S1.pdf]

Table S1. Multivariable logistic regression assessing factors associated with missing serotype data among invasive pneumococcal disease (IPD) cases, Italy, 2018–2023.

Variable	Level	N observations	N missing	% missing	OR	95% CI	p-value
**Age class**	0	140	57	40.7	1.00	Reference	—
	1–4	169	48	28.4	1.69	1.03–2.79	0.038
	5–9	90	41	45.6	0.81	0.46–1.43	0.471
	10–14	54	26	46.3	0.90	0.46–1.77	0.751
	15–24	78	28	34.6	1.33	0.73–2.46	0.349
	25–64	2354	926	39.3	0.99	0.69–1.43	0.977
	>64	4187	1516	36.2	1.01	0.70–1.45	0.944
**Time period**	2018–19	3226	1240	38.4	1.00	Reference	—
	2020–21	1005	526	52.1	0.56	0.48–0.64	<0.001
	2022–23	2841	876	30.8	1.43	1.28–1.60	<0.001
**Macro area**	Central	751	399	53.0	1.00	Reference	—
	Northern	5961	1980	33.2	2.36	2.01–2.76	<0.001
	Southern/Islands	360	263	72.5	0.43	0.33–0.56	<0.001
**(Intercept)**	—	—	—	—	0.80	0.55–1.18	0.257

The dependent variable was serotype availability (available = 1, missing = 0); predictors included age class, time period, and macro-area. ORs > 1 indicate higher odds of serotyping completeness.
