# Supplementary figures and images for: Invasive Pneumococcal Diseases Before and After the COVID-19 Pandemic in Italy (2018–2023)
Source: Microorganisms. 2025 Nov 30;13(12):2734. doi: 10.3390/microorganisms13122734 (PMC12735837; doi:10.3390/microorganisms13122734)

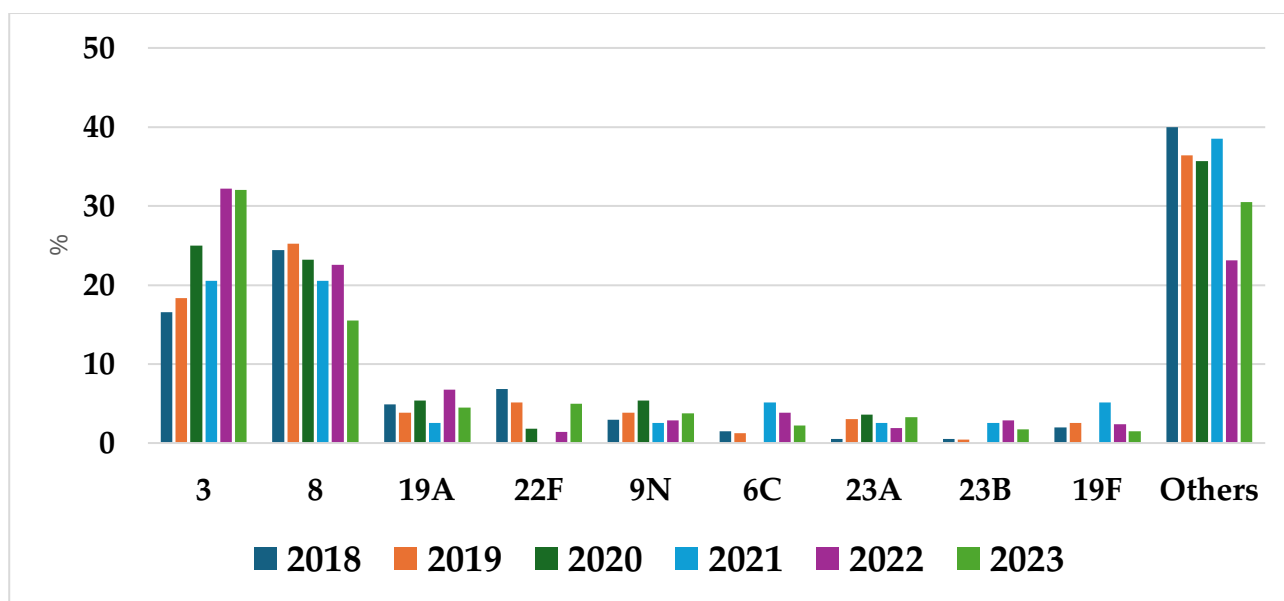

Figure S1. Serotype distribution in pneumococcal pneumonia in adults > 64 years of age, 2018–2023.

Supplement: Supplementary file 1 [file microorganisms-13-02734-s001.zip › Supplementary_Materials_Figure S1.pdf]

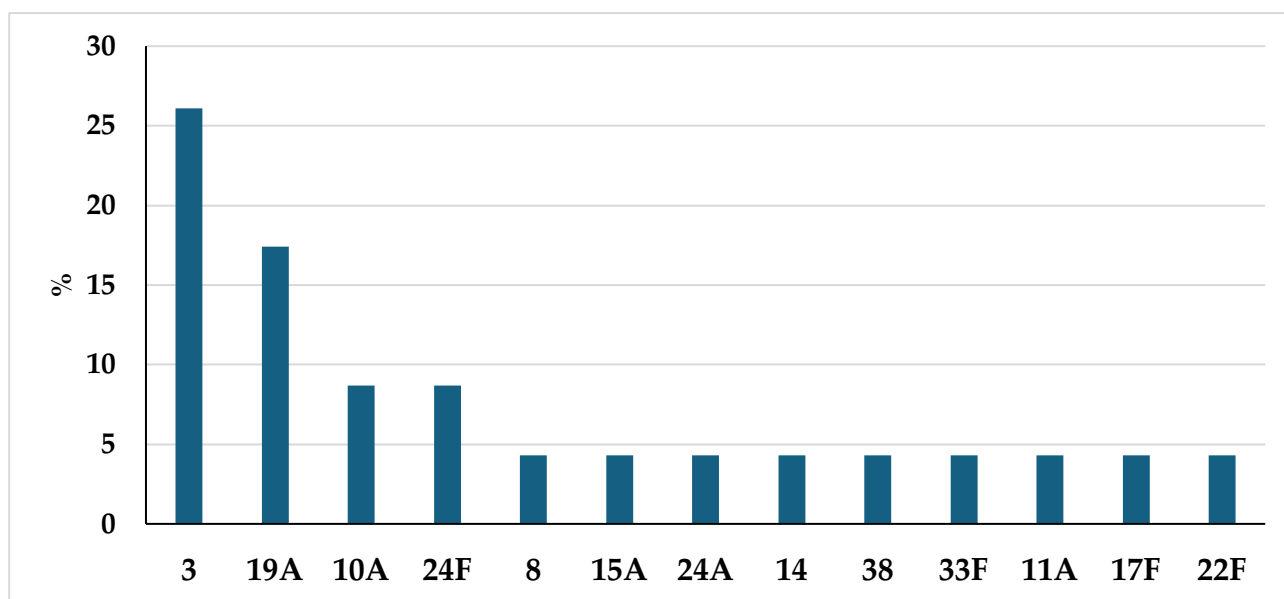

Figure S2. Serotype distribution in pneumococcal pneumonia in children <5 years of age in 2023.

Supplement: Supplementary file 1 [file microorganisms-13-02734-s001.zip › Supplementary_Materials_Figure S2.pdf]

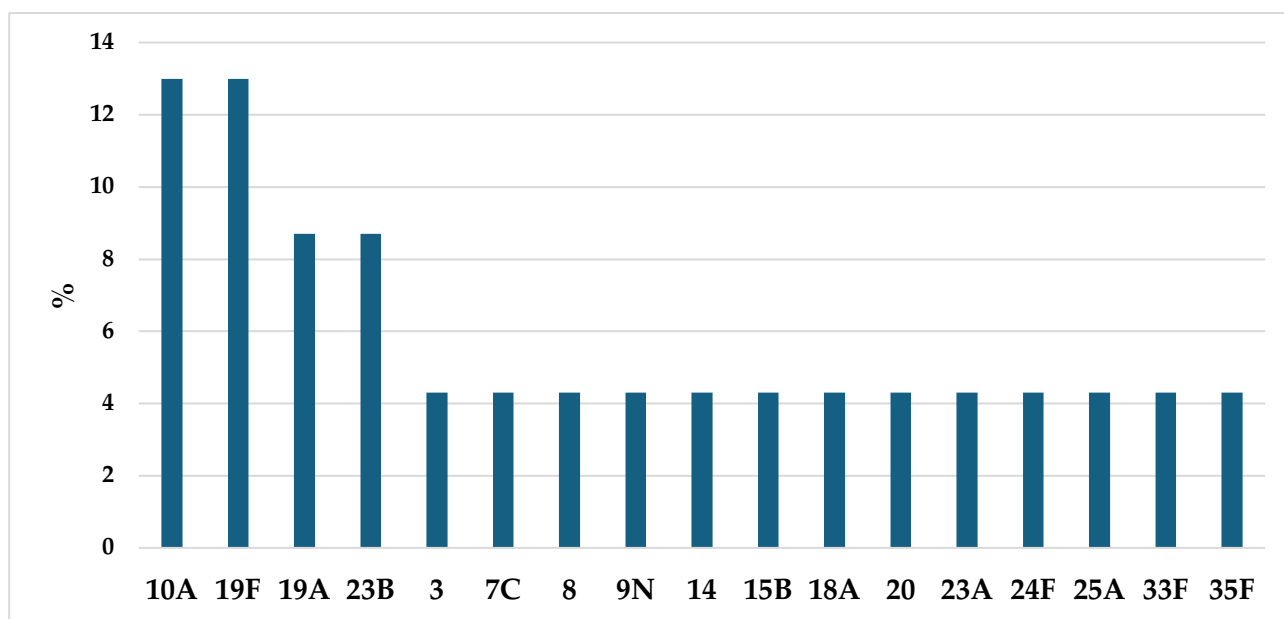

Figure S3. Serotype distribution in pneumococcal meningitis in children <5 years of age, 2022–2023.

Supplement: Supplementary file 1 [file microorganisms-13-02734-s001.zip › Supplementary_Materials_Figure S3.pdf]

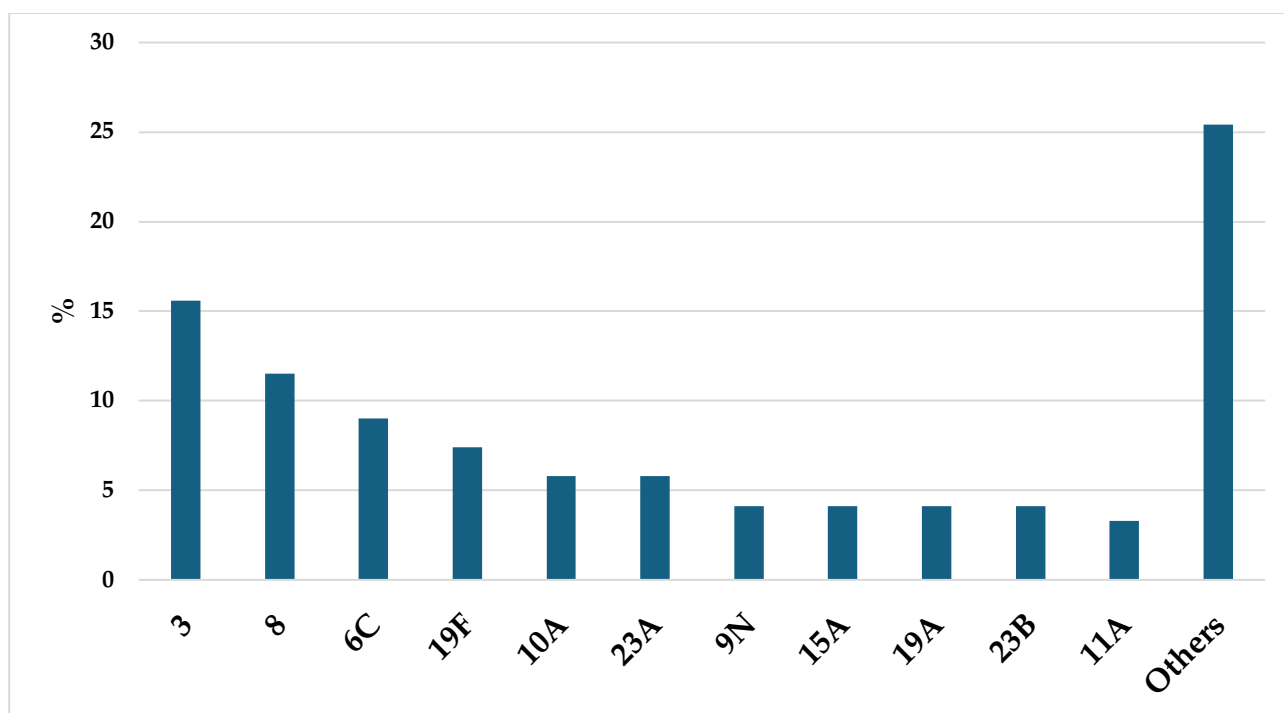

Figure S4. Serotype distribution in pneumococcal meningitis in adults >64 years of age, 2022–2023.

Supplement: Supplementary file 1 [file microorganisms-13-02734-s001.zip › Supplementary_Materials_Figure S4.pdf]

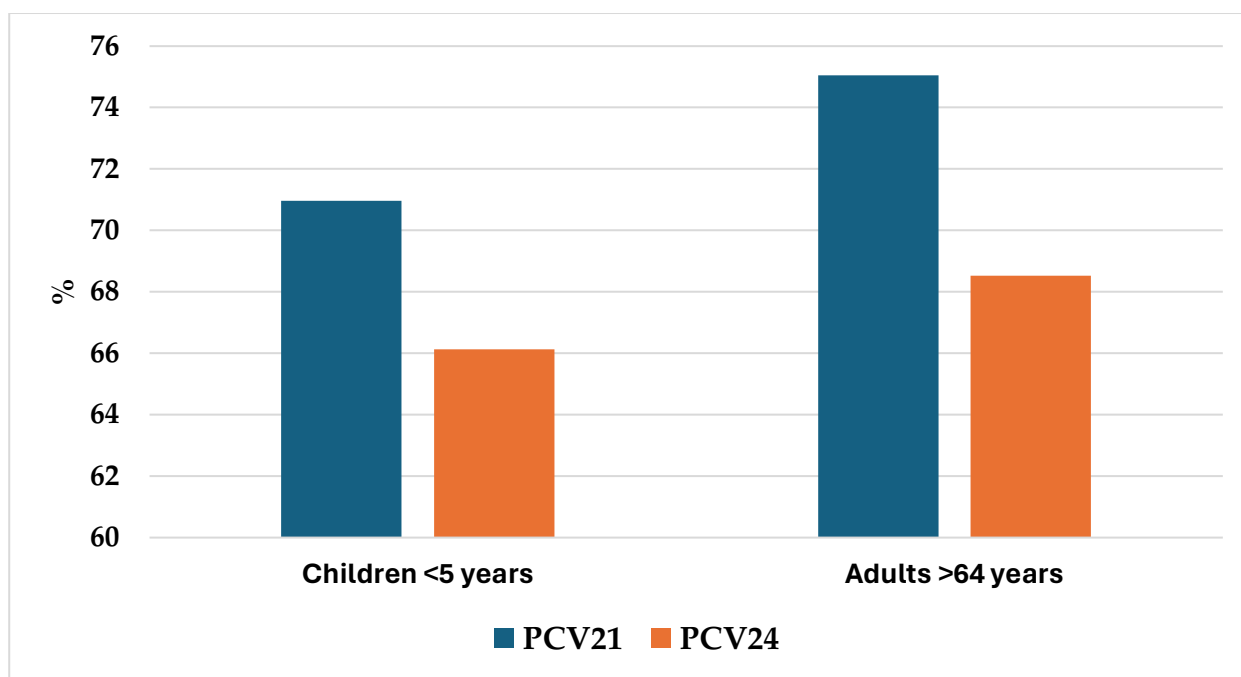

**Figure S5.** Serotype distribution by PCV21 and PCV24 in IPD in children <5 years and adults >64 years old in 2023.

Supplement: Supplementary file 1 [file microorganisms-13-02734-s001.zip › Supplementary_Materials_Figure S5.pdf]
